# Robust differential gene expression patterns in the prefrontal cortex of male mice exposed to an occupationally relevant dose of laboratory-generated wildfire smoke

**DOI:** 10.1093/toxsci/kfae097

**Published:** 2024-08-06

**Authors:** Adam Schuller, Jessica Oakes, Tom LaRocca, Jacqueline Matz, Matthew Eden, Chiara Bellini, Luke Montrose

**Affiliations:** Department of Environmental and Radiological Health Sciences, Colorado State University, Fort Collins, CO 80523, United States; Department of Bioengineering, Northeastern University, Boston, MA 02120, United States; Department of Health and Exercise Science, Colorado State University, Fort Collins, CO 80523, United States; Department of Bioengineering, Northeastern University, Boston, MA 02120, United States; Department of Bioengineering, Northeastern University, Boston, MA 02120, United States; Department of Bioengineering, Northeastern University, Boston, MA 02120, United States; Department of Environmental and Radiological Health Sciences, Colorado State University, Fort Collins, CO 80523, United States

**Keywords:** wildfire smoke, wildland firefighter, occupational health, transcriptomics

## Abstract

Wildfires have become common global phenomena concurrent with warmer and drier climates and are now major contributors to ambient air pollution worldwide. Exposure to wildfire smoke has been classically associated with adverse cardiopulmonary health outcomes, especially in vulnerable populations. Recent work has expanded our understanding of wildfire smoke toxicology to include effects on the central nervous system and reproductive function; however, the neurotoxic profile of this toxicant remains ill-explored in an occupational context. Here, we sought to address this by using RNA sequencing to examine transcriptomic signatures in the prefrontal cortex of male mice modeling career wildland firefighter smoke exposure. We report robust changes in gene expression profiles between smoke-exposed samples and filtered air controls, evidenced by 2,862 differentially expressed genes (51.2% increased). We further characterized the functional relevance of these genes highlighting enriched pathways related to synaptic transmission, neuroplasticity, blood–brain barrier integrity, and neurotransmitter metabolism. Additionally, we identified possible contributors to these alterations through protein–protein interaction network mapping, which revealed a central node at ß-catenin and secondary hubs centered around mitochondrial oxidases, the Wnt signaling pathway, and gene expression machinery. The data reported here will serve as the foundation for future experiments aiming to characterize the phenotypic effects and mechanistic underpinnings of occupational wildfire smoke neurotoxicology.

The wildfire landscape is rapidly changing across the globe with fires burning more intensely and for longer periods of time throughout the year, a trend which is expected to continue with warmer and drier climates (Higuera et al. 2023; Turco et al. 2023). In the United States, this has significantly increased the contribution of wildfire smoke to ambient air pollution in recent years (Childs et al. 2022). Although wildfire smoke is a complex mixture of chemical constituents that differ based on conditions surrounding each burn, it consistently comprised the largest percent of mass by volume particulate matter which is 2.5 microns or smaller in aerodynamic diameter (PM_2.5_) (Kim et al. 2018). This size fraction of air pollution particulate matter is especially important in a toxicologic context due to the fact that its exposure has been correlated with many adverse human health outcomes (Reid et al. 2016; Reid and Maestas 2019; Thangavel et al. 2022). Importantly, recent work has reported greater respiratory hospitalization incidence in individuals exposed to wildfire smoke PM_2.5_ when compared with air pollution from other sources (Aguilera et al. 2021). In addition to aberrancies in the cardiopulmonary system, wildfire smoke exposure has been increasingly associated with cognitive decline and reproductive dysfunction (Rubin et al. 2021; Cleland et al. 2022; Wen and Burke 2022). Still, these outcomes remain under-investigated in populations of individuals exposed to higher concentrations of wildfire smoke in an occupational setting (i.e. wildland firefighters).

Wildland firefighters are subjected to extreme and adverse work conditions throughout their shifts while performing diverse tasks (Adetona et al. 2017; Navarro et al. 2021a, 2021b; Ruby et al. 2023). These exposures have been associated with negative acute cardiac and respiratory health outcomes, impaired kidney performance, and hearing loss (Adetona et al. 2016; Navarro et al. 2019; Wu et al. 2021); however, emphasis on long-term disease risk has recently been identified as a major concern in this population, especially in regard to the central nervous system (CNS) (Pelletier et al. 2022). Our group has previously reviewed the literature linking wildfire smoke exposure to Alzheimer’s disease (AD) dementia (Schuller and Montrose 2020). Further, concentrated ambient wildfire smoke exposure in mice, mimicking a public health-relevant dose, has recently been shown to result in neuroinflammation and neurometabolic consequences using targeted molecular approaches in the prefrontal cortex (PFC) (Scieszka et al. 2022). We have demonstrated our ability to model occupational wildfire smoke exposure using a murine model in a laboratory environment (Schuller et al. 2021; Eden et al. 2023). Here, we sought to explore the effects of career wildland firefighter biomass combustion smoke exposure on the PFC transcriptome in male mice using RNA sequencing (RNAseq), an unbiased technique to quantify gene expression changes.

## Methods

### Animals

#### Ethics statement

All experiments performed using animal models were conducted in accordance with NIH guidelines under protocols approved by the Institutional Animal Care and Use Committee (IACUC) at Northeastern University. Eight-wk-old male Apoe^−/−^ mice, bred on a C57BL/6 background, were obtained from Jackson Laboratory (Bar Harbor, ME, United States). Apoe^−/−^ mice represent an environmentally susceptible strain due to their impaired lipid metabolism and propensity to develop atherosclerosis (Yamashita et al. 2014). This strain has also been utilized to characterize the infiltration of ultrafine particulate matter into the CNS (Peters et al. 2006). Importantly, even at aged time points these mice do not exhibit neuro-pathology consistent with AD dementia (Ganor et al. 2018). All mice were housed in clear plastic cages, in groups of up to 5, with ad libitum food and water. Cages were housed in a temperature-controlled vivarium on a 12 h light/dark cycle.

#### Experimental design

Eight-wk-old male Apoe^−/−^ mice were randomly assigned to one of two groups: Smoke-exposed or filtered air control (*n* = 8 mice/group). Mice were exposed to filtered air or simulated wildfire smoke in a 3D printed exposure chamber, which has been experimentally validated to evenly distribute particulate matter (Eden et al. 2023), for 2 h/d, 5 d/wk, for 16 wk. Simulated wildfire smoke was generated using a quartz-tube and ring furnace setup mounted on a linear actuator, as previously described (Garg et al. 2021). Target smoke concentration was set to 40 mg/m^3^ and maintained by diluting the simulated smoke with filtered indoor air. This dose and duration of exposure were determined to be occupationally relevant based on calculations of deposited particulate mass in the lungs of representative mice and men, normalized by lung surface area in the two species, as described previously (Eden et al. 2023). The average total PM concentration across the duration of the simulated smoke exposure was 39 ± 13 mg/m^3^ and the average CO concentration across the 16 wk was 218 ± 46 ppm. Extensive characterization of the toxicologic profile of this smoke has been previously reported (Garg et al. 2021; Eden et al. 2023). All mice were monitored for signs of overt toxicity and all mice survived for the duration of the study with a final body mass percent change of 24 ± 1% for the filtered air controls and 19 ± 1% for the smoke-exposed animals across the 16-wk study. Carboxyhemoglobin levels were measured across the study and are reported to be 1.7 ± 0.9% for the filtered air control group and 21.2 ± 4.8% for the smoke-exposed group. Mice were sacrificed within 48 h of the final day of exposure at Northeastern University and tissue was dissected, flash frozen, and shipped to Boise State University for storage at −80°C (*n* = 8 mice/group).

### RNA sequencing

#### Library preparation and sequencing

Approximately 30 to 35 mg of microdissected PFC per sample (*n* = 8 mice/group) were RNA extracted and sequenced by technicians at Novogene (Beijing, China). Samples were subjected to quality control via nanodrop spectroscopy and agarose gel electrophoresis. Subsequently, mRNA was enriched using oligo(dT) beads, fragmented, and converted to cDNA using random hexamer primers via RT–PCR following a standard protocol. Library quality control was assessed using a Qubit fluorometer and libraries were diluted to 1 ng/µl concentration before insert size distribution was checked using Agilent 2100. Clustered libraries were sequenced using the Illumina HiSeq platform generating 150 bp paired-end reads.

#### Analysis workflow

Raw data (∼54,750,000 reads/sample) were cleaned using fastp software by removing reads with adapter contamination, greater than 10% uncertain nucleotides, or base quality <5 for more than 50% of the read. Clean reads (∼54,500,000/sample) were aligned to the mouse reference genome using Hisat2 v2.0.5 (96.7% total mapping efficiency) and assembled by StringTie v1.3.3b. Fragments per kilobase per million base pairs sequenced (FPKM) were calculated using featureCounts v1.5.0-p3. Gene expression analysis of smoke-exposed versus filtered air control groups was performed using the edgeR R package (v3.22.5) through one scaling normalized factor. Thresholds of false discovery rate (FDR) <0.05 and log2foldchange were used to determine differentially expressed genes (DEGs). DEGs were then analyzed for Gene Ontology (GO), Kyoto Encyclopedia of Genes and Genomes (KEGG), and Reactome pathways using the clusterProfiler R package, correcting for gene length bias. Significance was determined by FDR <0.05 when comparing between exposure groups. GO pathways enriched for biological process (BP) or cellular component (CC) classifications between groups were subsequently visualized using REVIGO (Supek et al. 2011). Systems-level visualization of DEGs was accomplished via the construction of protein–protein interaction (PPI) networks via the STRING database plug-in for Cytoscape, including known and predicted interactions. To visualize these networks, we applied the Fruchterman–Reingold force-directed layout in Gephi (v0.10.1). Functional modules were further defined (*n* = 3 clusters) using the K means algorithm, with the top GO BP term being used to classify each cluster (FDR<0.05) via iDEP.96 (Ge et al. 2018).

## Results

### Overall transcriptional profile of wildfire smoke-exposed and filtered air control samples

**Fig. 1.**
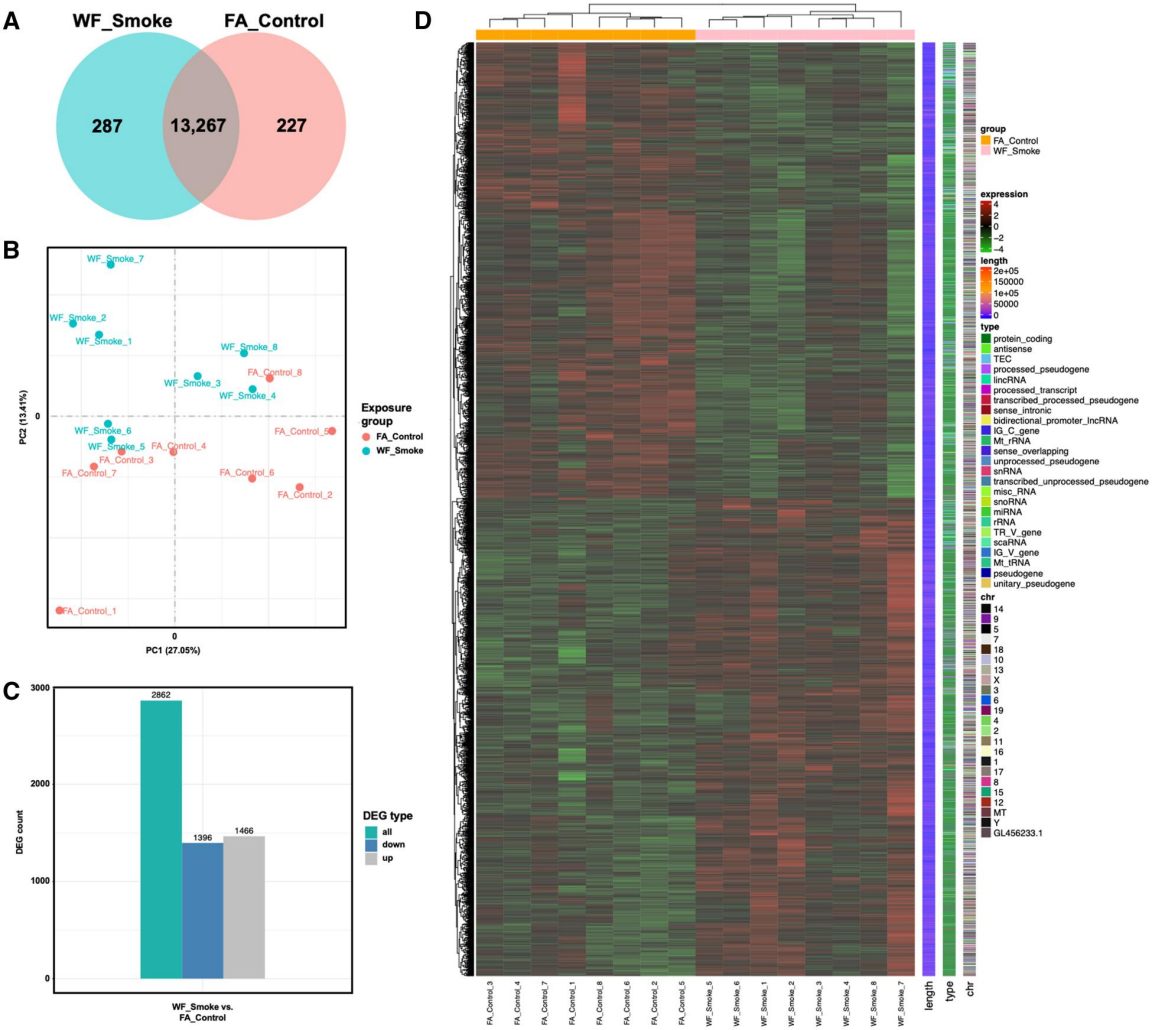
A) Venn diagram demonstrating overlap in the genes mapped to reads for each treatment group. Two hundred twenty-seven and 287 genes were found to be uniquely enriched in filtered air control or wildfire smoke-exposed cohorts, respectively. B) PCA plot comparing the variance of principal components 1 and 2 between overall gene expression profiles of all samples across both experimental groups. PCA analysis revealed distinct clustering by group. C) Bar graph highlighting the differentially expressed genes (DEGs) between smoke-exposed and filtered air control groups identified using edgeR (FDR<0.05 and log2foldchange). One thousand three hundred ninety-six genes were found to be decreased and 1,466 were found to be increased between groups. D) Heatmap generated by unsupervised clustering of genes with the highest variation across the dataset. Transcriptional profile of smoke-exposed samples is distinct when compared with filtered air control samples.

To begin to identify the effects of wildfire smoke exposure in the PFC of male mice, we utilized RNA-seq to compare gene expression profiles of smoke-exposed animals relative to filtered air controls (*n* = 8/group). Across all samples, 93.75% of reads were found to be in exon regions which mapped to a total of 13,781 genes. Two hundred eighty-seven (2.1%) of those genes were found to be unique to wildfire smoke samples, whereas 227 (1.6%) genes were unique to filtered air controls (Fig. 1A). In order to assess variance across the sample population, we generated a PCA plot using the gene expression value (FPKM) of all samples (Fig. 1B) which demonstrated two distinct clusters, one encompassing the wildfire smoke treated samples and the other containing the filtered air controls. DEG analysis revealed that 2,862 genes were differentially expressed between groups, with 1,396 genes/transcripts increased and 1,466 decreased in wildfire smoke-exposed samples compared with filtered air controls (Fig. 1C). We additionally performed a hierarchical clustering analysis of the FPKM values of DEGs across the dataset to examine the effects of smoke exposure on transcriptional patterns (Fig. 1D) which demonstrated distinct clustering of samples by exposure group and broad distribution of DEGs across the genome.

**Fig. 2.**
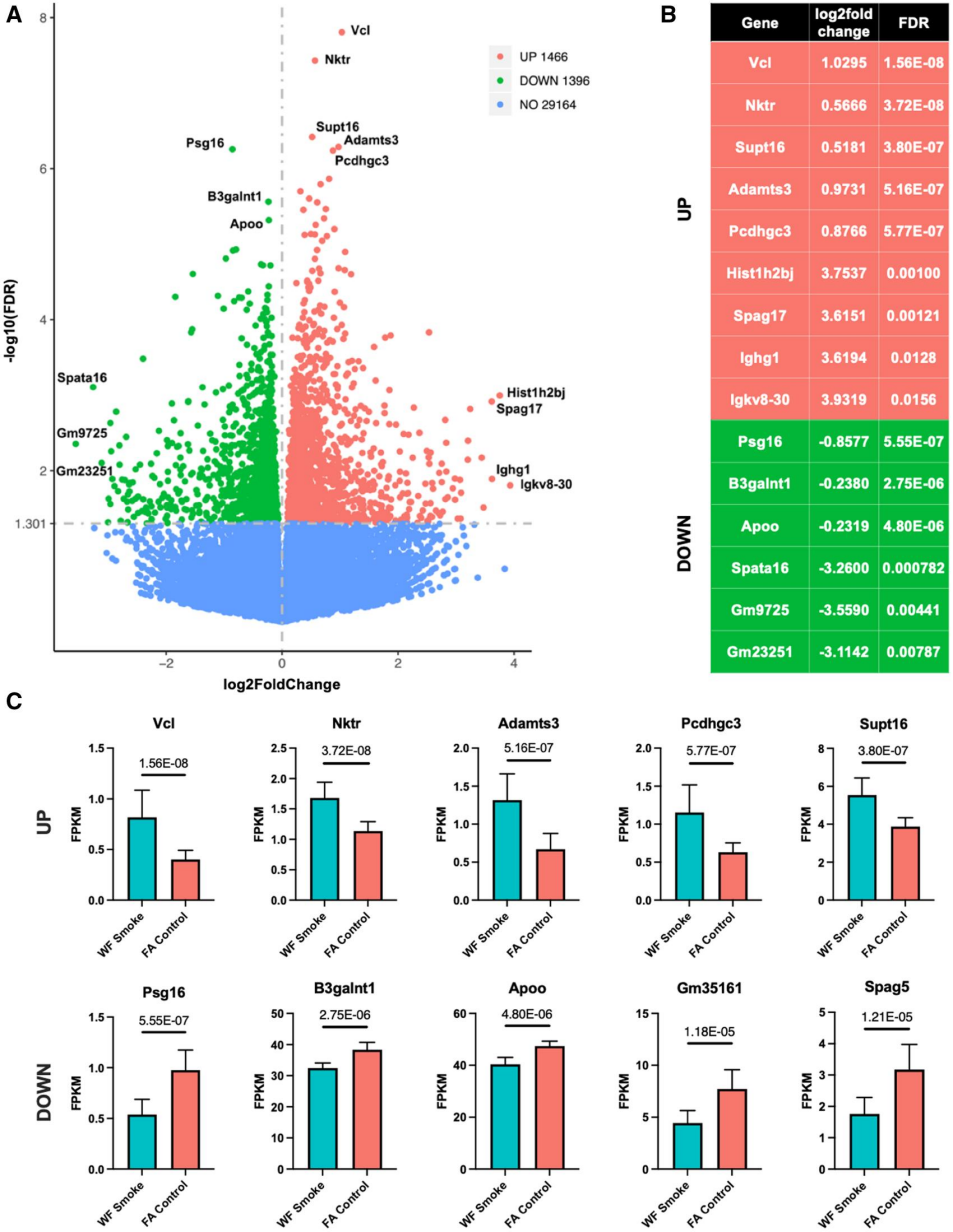
A) Volcano plot demonstrating expression of all of the genes mapped from the dataset. Annotations for the top genes (up and down) by strongest FDR and/or log2foldchange. A) Expression data for the top genes annotated in volcano plot in panel (A). C) Bar graphs depicting the FPKM value (mean±SD) of each of the top 5 increased (top) and decreased (bottom) genes by strongest FDR. Abbreviations: Vinculin (*Vcl*), natural killer tumor recognition sequence (*Nktr*), suppressor of Ty 16 (*Supt16*), a disintegrin-like and metallopeptidase (resprolysin type) with thrombospondin type 1 motif 3 (*Adamts3*), protocadherin gamma subfamily C 3 (*Pcdhgc3*), pregnancy specific glycoprotein 16 (*Psg16*), ß-1,3-galactosaminyltransferase polypeptide 1 (*B3galnt1*), apolipoprotein O (*Apoo*), predicted gene 35161 (*Gm35161*), sperm associated antigen 5 (*Spag5*). Value over each comparison represents FDR.

To further characterize the effects of wildfire smoke exposure on specific genes across the transcriptome, we identified top DEGs across the dataset. Figure 2A shows a volcano plot with all mapped genes across the dataset. DEGs were identified as genes with log2foldchange and FDR<0.05. Selected top hits were binned by increased or decreased expression and strength of log2foldchange or FDR (Fig. 2B). We also represent the FPKM of each of the top 5 strongest (by FDR) increased and decreased genes/transcripts (Fig. 2C). The most strongly increased genes included: Vinculin (*Vcl*), natural killer tumor recognition sequence (*Nktr*), suppressor of Ty 16 (*Supt16*), a disintegrin-like and metallopeptidase (resprolysin type) with thrombospondin type 1 motif 3 (*Adamts3*), and protocadherin gamma subfamily C 3 (*Pcdhgc3*). Conversely, the most strongly decreased genes included: Pregnancy specific glycoprotein 16 (*Psg16*), ß-1,3-galactosaminyltransferase polypeptide 1 (*B3galnt1*), apolipoprotein O (*Apoo*), predicted gene 35161 (*Gm35161*), and sperm associated antigen 5 (*Spag5*). The relevance of these genes to the PFC and CNS disease states will be further explored in the discussion.

### Functional enrichment analysis

**Fig. 3.**
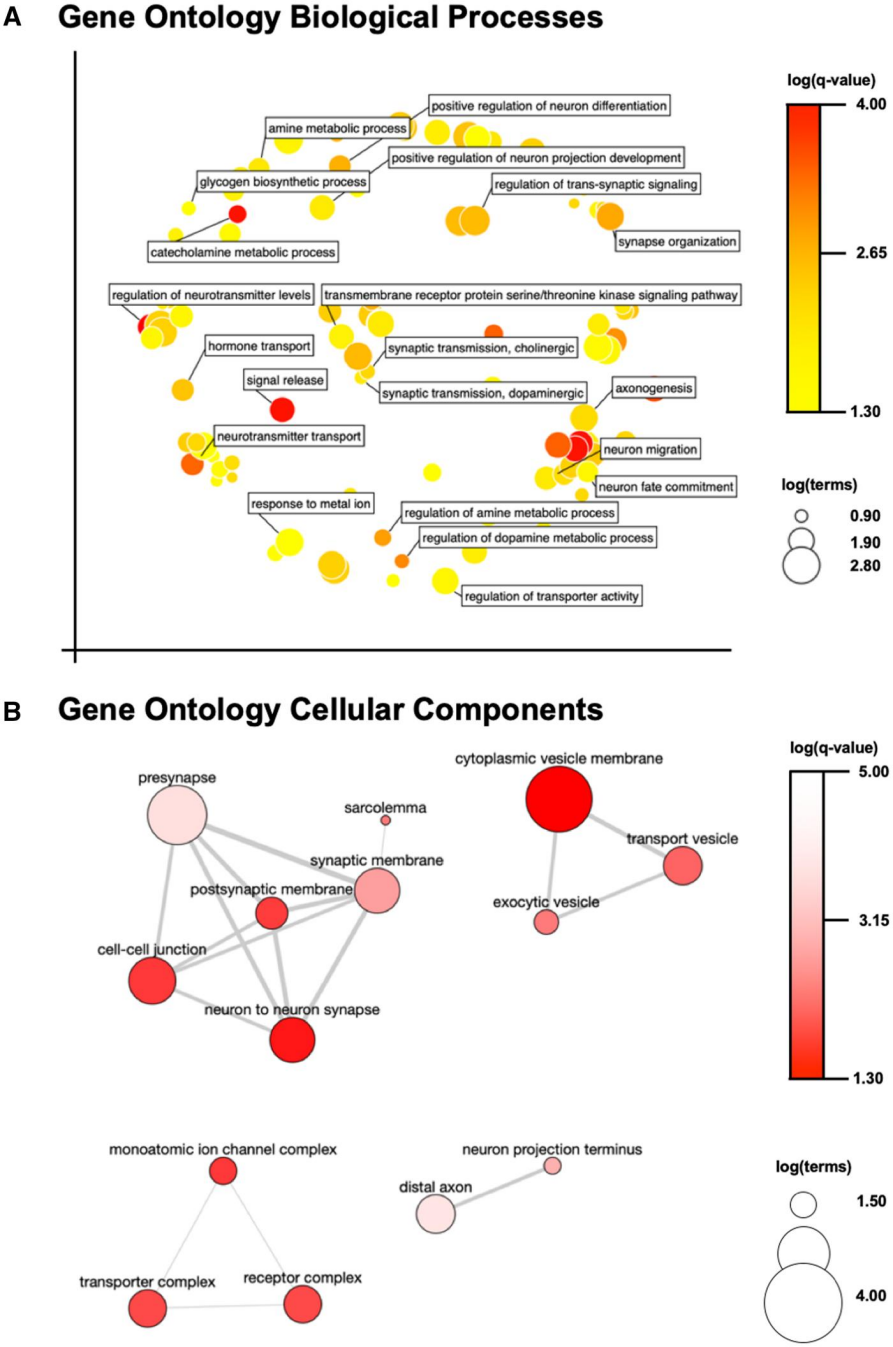
A) Multidimensional scaling plot representing the top gene ontology (GO) biological process (BP) terms by semantic similarity which are enriched between smoke-exposed and filtered air control samples (*q*-value<0.05) across all 2,862 DEGs identified in our dataset. B) Pathway interaction map highlighting GO pathways in the cellular component (CC) category between smoke-exposed and filtered air control samples (*q*-value<0.05) across the entire DEG dataset. For both panels, color scale corresponds to log(*q*-value), whereas symbol size corresponds to log(no. of terms).

**Fig. 4.**
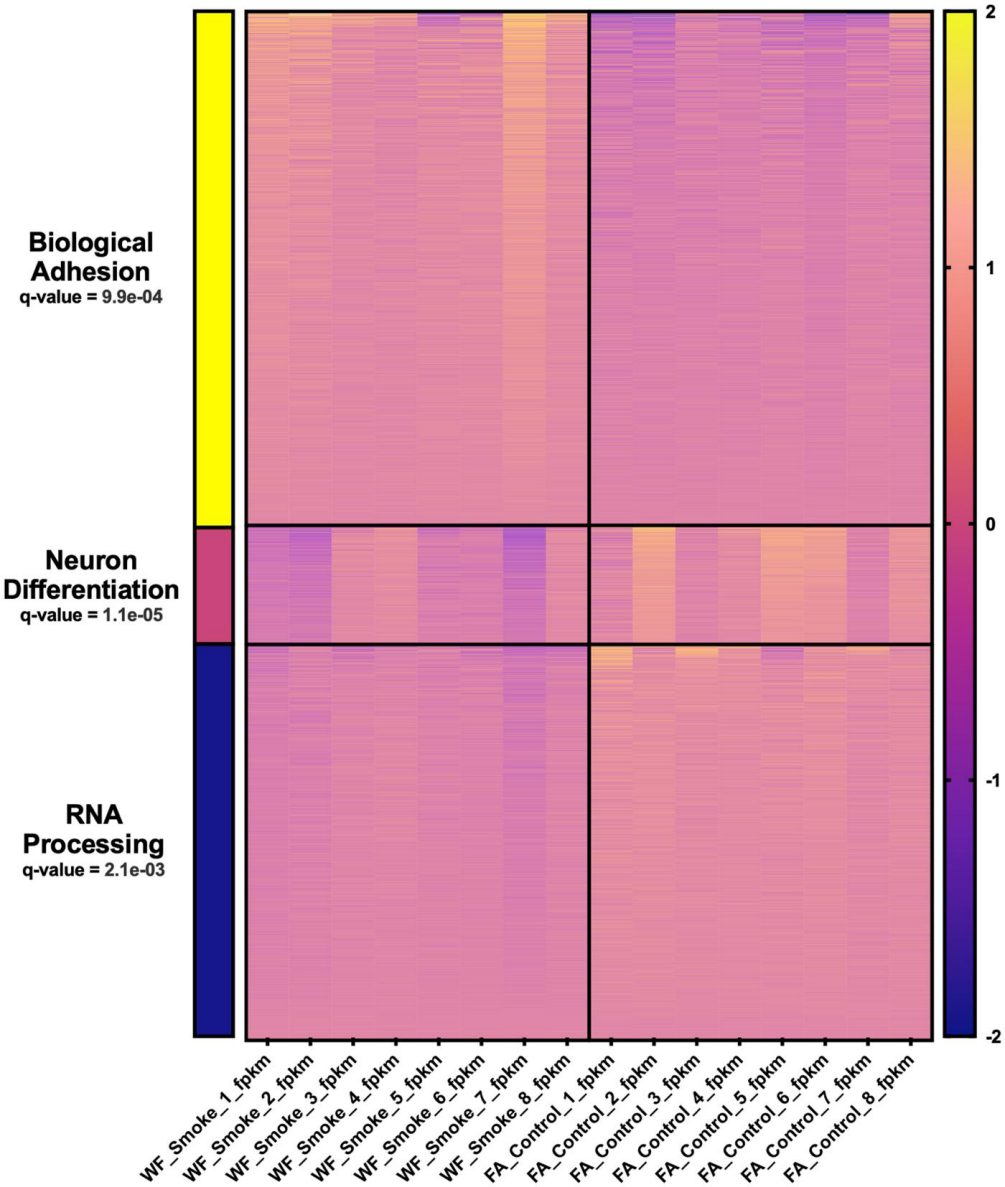
Heatmap depicting results of the k-means clustering analysis (*n* = 3 clusters) for all 2,862 DEGs using iDEP.96. Top gene ontology (GO) biologic process (BP) term used to classify each cluster (FDR<0.05) as associated with cluster bar color on the left side of the image: Biological adhesion (yellow), neuron differentiation (pink), and RNA processing (blue). Color scale on the right side of the image represents the log-transformed FPKM value of each gene/transcript.

**Fig. 5.**
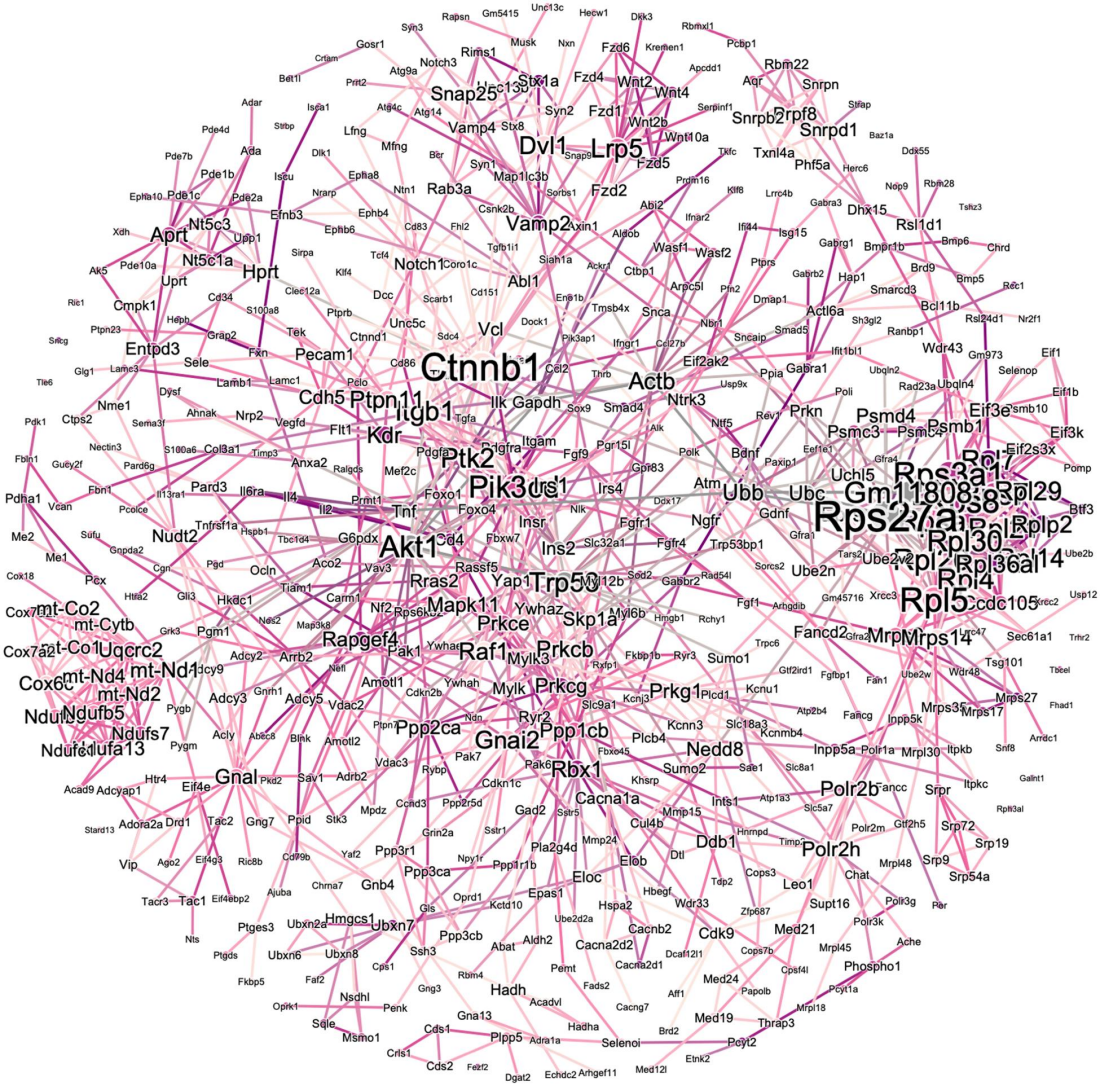
Protein–protein interaction (PPI) network map assembled using STRING database plug-in for cytoscape and visualized using Gephi (Freuchterman–Reingold) for the 2,862 DEGs identified in our dataset between smoke-exposed and filtered air controls. The size of each node corresponds to the degree and the color of each edge corresponds to the string strength score (integrating known and predicted interactions).

In order to better understand the functional trends of DEGs across the dataset, we employed multiple pathways analyses. Firstly, GO terms were assessed across the entire set of 2,862 DEGs. Pathways relevant to neuronal function (Fig. 3A, e.g. metabolic processes, neurotransmitter synthesis/release, synaptic transmission) as well as neuronal compartmentalization (Fig. 3B, e.g. presynapse, cytoplasmic vesicle membrane, synaptic membrane) were found to be most strongly enriched between groups. This was additionally confirmed by k-means clustering which distinctly identified 3 clusters with Biological Adhesion, Neuron Differentiation, and RNA Processing as the most strongly associated GO BP terms (Fig. 4). To further assess functional enrichment across the dataset, we analyzed the KEGG and Reactome pathways associated with either the 1,396 decreased DEGs (Fig. S1A and C) or the 1,466 increased DEGs (Fig. S1B and D) uniquely. The top 5 strongest KEGG pathways associations across either dataset were: Synaptic vesicle cycle (down), neuroactive ligand–receptor interaction (up), insulin secretion (down), retrograde endocannabinoid signaling (down), and oxidative phosphorylation (down). The five strongest Reactome pathways associations across either dataset were: Amine ligand-binding receptors (up), neuronal system (down), transmission across chemical synapses (down), serotonin neurotransmitter release (down), and glutamate neurotransmitter release (down). Lastly, we visualized the PPI network map generated using the STRING plug-in for cytoscape via Gephi. This resulted in a network (Fig. 5) with a central node at ß-catenin and secondary hubs around gene expression machinery, Wnt signaling, and mitochondrial oxidases.

## Discussion

With warmer and dryer climates increasing wildfire prevalence worldwide, smoke exposure is of increasing concern regarding public and occupational health, including recently associated adverse CNS outcomes (Oudin et al. 2018; Sosedova et al. 2021; Scieszka et al. 2022, 2023; Zhang et al. 2023). To address this, we explored the effects of chronic exposure to laboratory-generated wildfire smoke, estimated to be equivalent to a career wildland firefighter exposure, on the gene expression profile of the PFC in male mice using RNA-seq. We report robust changes in gene expression across the genome and functionally characterized DEGs between groups using a battery of bioinformatic approaches. Here, we will discuss the contextual relevance of those disruptions given our experimental paradigm and previously published work.

The PFC was selected as the brain region of interest due to its dynamic involvement in development and cognitive function (Koechlin 2016; Xu et al. 2019; Kolk and Rakic 2022), its high level of conservation between rodents and humans (Chini and Hanganu-Opatz 2021), and its documented susceptibility to environmental toxicant exposure (Tooley et al. 2021). Human PFC has been shown to be reduced in volume following ambient PM_2.5_ exposure (Gale et al. 2020; Petkus et al. 2022). Further, both male and female mice have been demonstrated to possess neuroinflammatory and neurometabolic phenotypes following public health-relevant doses of wildfire smoke or biomass combustion smoke exposure, respectively (Scieszka et al. 2022, 2023). Here, our results indicate that there are robust trends of dysregulation in synaptic transmission (Fig. 3), and suggest specific alterations in serotonergic, cholinergic, and dopaminergic neurotransmitter metabolism pathways following chronic occupational smoke exposure (Fig. 3 and Fig. S1). This is consistent with previous work demonstrating lower serotonergic metabolite levels in human urine following occupational air pollution exposure (Tomei et al. 2004) as well as dysregulation of serotonin across the limbic system in both mice and rats exposed to ambient air pollution (Mokoena et al. 2015; Yokota et al. 2016). Atypical serotonin signaling in the PFC has been broadly reviewed previously (Puig and Gulledge 2011), highlighting the involvement of this disrupted transmission in aberrant neuropsychiatric outcomes. It is worth noting that increased prevalence of PTSD, suicide risk, and other neuropsychiatric symptoms have been reported in career wildland firefighters and in volunteer firefighters taking part in wildfire containment efforts (Stanley et al. 2018; Schnell et al. 2020). Although behavioral assessment was not included in our study, previous groups have demonstrated the ability of biomass combustion smoke to drive aberrant exploratory behavior, locomotor patterns, spatial navigation, and anxiety even into subsequent generations of paternally exposed rats (Sosedova et al. 2021). This locomotor disruption is especially interesting in the context of the differential cholinergic and dopaminergic metabolic pathways enrichment in our current study (Fig. 3A), because the recruitment of both of these neurotransmitters in the PFC has been shown to modulate movement governing signaling in the striatum (Adrover et al. 2020).

Exposure to wildfire smoke has been recently associated with cognitive dysfunction, including a significantly heightened risk of AD dementia in a human epidemiologic cohort when compared with other air pollutants (Zhang et al. 2023). Further, exposures commonly faced during wildland firefighting tasks have also been recently correlated with cognitive decline and dementia risk (Genuis and Kelln 2015). Although the mechanism underlying the aforementioned associations is not well understood, our study highlights functionally enriched pathways and specific gene targets to explore in this context. In our present dataset, the transcriptomic profile is highly similar to those previously identified in AD (Nativio et al. 2020), advanced chronologic age (Dillman et al. 2017; Mohan et al. 2018), and accelerated aging cohorts (Cavalier et al. 2021). This is reflected by the differential enrichment for KEGG pathways including Huntington’s disease, Parkinson’s disease, and AD in this dataset (Fig. S1). Mechanistically, one term that stands out in our dataset in the context of AD pathogenesis is the serine/threonine kinase signaling pathway (Fig. 3A). These kinases are directly responsible for the phosphorylation of microtubule-associated protein tau in AD, as well as other tauopathies, and contribute to pathogenic aggregation and subsequent neuronal loss (Perluigi et al. 2016). Further, ß-catenin (*Ctnnb1*) presents as a central node in our PPI network map (Fig. 5). This protein is known to modulate neuroplasticity (Maguschak and Ressler 2012) and, when activated, has been shown to curb blood–brain barrier (BBB) dysfunction (Wang et al. 2022). Similarly, dysregulation of ß-catenin has been implicated in accelerated aging and AD (Palomer et al. 2019). Strongly associated with this central node, we also see enrichment of the Wnt signaling pathway (Fig. 5). Interestingly, the Wnt/ß-catenin signaling pathway has been previously demonstrated to be enriched in an analysis of the effects of wildfire smoke on the nonhuman primate nasal epigenome (Brown et al. 2022). This signaling pathway has also been implicated in the pathogenesis of tauopathies and AD (Dengler-Crish et al. 2018). In the same PPI network in our present study, we report a hub centered around mitochondrial oxidases (Fig. 5). Mitochondrial dysfunction has been previously reported in the male rat brain following exposure to smoldering biomass combustion smoke (Lee et al. 2010) and aberrant metabolomic profiles have been reported in the hippocampus of female mice exposed to smoke from wood chip combustion (Scieszka et al. 2023). Mitochondrial dysfunction has further been implicated in the pathogenesis of AD, and is currently being explored as a potential druggable target in this disease (Bhatia et al. 2022). Given the dynamic nature of these previous reports and our work, it is necessary to further explore the effects of duration of exposure to wildfire smoke, as well as to expand the assessment strategy to include additional brain regions. It is also worth examining these molecular cascades at a cellular resolution either by implementing single-cell transcriptomic approaches or by assessing protein expression with histopathologic techniques (i.e. immunofluorescent imaging modalities).

On a gene-specific basis, vinculin (*Vcl*) was found to be the most significantly increased in smoke-exposed compared with filtered air control samples (Fig. 2). Interestingly, vinculin levels have also been shown to increase in the PFC of rats exposed to a single prolonged stressor (Li et al. 2015). Further, MAPK-vinculin signaling has been shown to modulate extracellular matrix (ECM) stiffness in response to environmental signals (Garakani et al. 2017). ECM remodeling was also implicated in our k-means clustering analysis which revealed a major cluster around biological adhesion (Fig. 4). Wildfire smoke exposure has been shown to increase the expression of matrix metalloproteinases (MMPs) in guinea pigs, including those known to digest tight junctions at the BBB (MMP2/9) (Ramos et al. 2021). More broadly, ECM degradation at the neurovascular unit is hypothesized as a mechanism through which air pollution might bypass the BBB to reach the CNS (Liu et al. 2021). On the other hand, research has previously demonstrated that iron soot is capable of being taken up directly through olfactory nerve bundles and tracts (Hopkins et al. 2018). This is also interesting in the context of recent work demonstrating changes in DNA methylation patterns in the nasal epithelia following wildfire smoke exposure in nonhuman primates, even persisting long after exposure ceased (Brown et al. 2022). In our study, whereas the route of wildfire smoke exposure to the CNS was not assessed, we did see evidence of other epigenetic alterations such as the hub centered around transcriptional and translational machinery visible in the PPI network map (Fig. 5) and the k-means cluster centered around RNA processing (Fig. 4). Our group has also previously shown that exposure to simulated wildfire smoke at a lesser dose and duration is sufficient to significantly disrupt DNA methylation patterns in the germ cells of male mice (Schuller et al. 2021). Taken together, this warrants future assessment of epigenetic alterations both in the context of CNS exposure route and in a mechanistic capacity once the toxicant, or other factors produced following inhalation, reach the brain parenchyma.

Interestingly, there did not appear to be significant alterations in canonical neuroinflammatory pathways in our primary analyses. This was unexpected given the reports of robust neuroinflammation in prior smoke exposure studies (Scieszka et al. 2022, 2023). Still, there are a variety of differences between this previous work and our current experiment which might explain the lack of conserved pathways disruption. For example, the dose and duration of smoke exposure in our study are both much greater than in previous analyses. Additionally, the carbon monoxide levels mice were subjected to in this study are far greater than in prior reports. Although the COHb levels observed in the present work are below the threshold of CO toxicity (Palmeri and Gupta 2023), it is possible that this co-exposure did exacerbate the effects associated with wildfire smoke PM inhalation. Still, the levels of CO ppm exposed to animals in this study are within plausible exposure levels observed in wildland firefighters performing various occupational tasks (Cone et al. 2005; Semmens et al. 2021). The increased CO and PM levels exposed to mice in this study are a direct consequence of our goal to model longitudinal wildland firefighter service as opposed to acute public health exposure. Moreover, these previous studies have employed targeted approaches to measure neuroinflammatory gene expression (e.g. RT–qPCR) which do not survey pathway enrichment across the entirety of the transcriptome. It is well established in the literature that when neuroinflammation is induced by environmental toxicant exposure, there are temporal dynamics associated with various stages of activation and resolution of specific glial cell responses (Rocha et al. 2022). For these reasons, we did follow-up with a brief targeted assessment of specific proteins identified in previous work. Interestingly, this highlighted concordant shifts in directionality for each of the genes/transcripts differentially expressed in our study mirroring prior reports (Fig. S2). Additionally, one major node in the center of a hub on our PPI network map (Fig. 5), *Rps27a*, has been recently implicated in microglial activation in neurodegeneration via control of neuroinflammation (Khayer et al. 2020). Although we cannot confirm that this is representative of expression changes in glia specifically in our study, this does warrant future exploration of neuroinflammatory cascades following smoke exposure, especially at a cell-specific resolution, by more diverse time points, and across other brain regions of interest implicated in neurodegenerative disease pathogenesis.

## Conclusion

We have here demonstrated that exposure to an occupationally relevant dose of simulated wildfire smoke results in robust disruption of gene expression patterns in the male mouse PFC. Future work is necessary to explore the phenotypic relevance of these molecular changes, as well as to tease apart the effects of administering similar doses of smoke across different patterns of exposure duration and at varied combustion parameters. Further, the integration of this work with other occupationally relevant exposure types through the implementation of multi-hit models, and an exploration of the sex differences in this context, would better advance our understanding of the neurotoxic risks associated with conducting wildland firefighting duties. This initial report of functionally relevant gene expression differences will guide downstream mechanistic analyses which can now apply targeted, evidence-based approaches.

## Supplementary Material

kfae097_Supplementary_Data

## References

[kfae097-B1] Adetona AM , Adetona O, Gogal RM, DIaz-Sanchez D, Rathbun SL, Naeher LP. 2017. Impact of work task-related acute occupational smoke exposures on select proinflammatory immune parameters in wildland firefighters. J Occup Environ Med. 59(7):679–690. doi: 10.1097/JOM.0000000000001053 [accessed 2023 Apr 13]. https://pubmed.ncbi.nlm.nih.gov/28692002/. 28692002 PMC6810646

[kfae097-B2] Adetona O , Reinhardt TE, Domitrovich J, Broyles G, Adetona AM, Kleinman MT, Ottmar RD, Naeher LP. 2016. Review of the health effects of wildland fire smoke on wildland firefighters and the public. Inhal Toxicol. 28(3):95–139. doi: 10.3109/08958378.2016.1145771. 26915822

[kfae097-B3] Adrover MF , Shin JH, Quiroz C, Ferré S, Lemos JC, Alvarez VA. 2020. Prefrontal cortex-driven dopamine signals in the striatum show unique spatial and pharmacological properties. J Neurosci. 40(39):7510–7522. doi: 10.1523/JNEUROSCI.1327-20.2020. [accessed 2024 Jan 31]. https://www.jneurosci.org/content/40/39/7510. 32859717 PMC7511190

[kfae097-B4] Aguilera R , Corringham T, Gershunov A, Benmarhnia T. 2021. Wildfire smoke impacts respiratory health more than fine particles from other sources: observational evidence from Southern California. Nat Commun. 12(1):1493. doi: 10.1038/s41467-021-21708-0. 10.1038/s41467-021-21708-0. 33674571 PMC7935892

[kfae097-B5] Bhatia S , Rawal R, Sharma P, Singh T, Singh M, Singh V. 2022. Mitochondrial dysfunction in Alzheimer’s disease: opportunities for drug development. Curr Neuropharmacol. 20(4):675–692. doi: 10.2174/1570159X19666210517114016. [accessed 2024 Feb 2]. https://www.ncbi.nlm.nih.gov/pmc/articles/PMC9878959/. 33998995 PMC9878959

[kfae097-B6] Brown AP , Cai L, Laufer BI, Miller LA, LaSalle JM, Ji H. 2022. Long-term effects of wildfire smoke exposure during early life on the nasal epigenome in rhesus macaques. Environ Int. 158:106993. doi: 10.1016/J.ENVINT.2021.106993. [accessed 2023 Feb 3]. https://pubmed.ncbi.nlm.nih.gov/34991254/. 34991254 PMC8852822

[kfae097-B7] Cavalier AN , Clayton ZS, Hutton DA, Wahl D, Lark DS, Reisz JA, Melov S, Campisi J, Seals DR, LaRocca TJ. 2021. Accelerated aging of the brain transcriptome by the common chemotherapeutic doxorubicin. Exp Gerontol. 152:111451. doi: 10.1016/J.EXGER.2021.111451. [accessed 2024 Feb 2]. https://pubmed.ncbi.nlm.nih.gov/34147619/. 34147619 PMC8319121

[kfae097-B8] Childs ML , Li J, Wen J, Heft-Neal S, Driscoll A, Wang S, Gould CF, Qiu M, Burney J, Burke M. 2022. Daily local-level estimates of ambient wildfire smoke PM 2.5 for the contiguous US. Environ Sci Technol. 56(19):13607–13621. doi: 10.1021/acs.est.2c02934. [accessed 2024 Jan 30]. 10.1021/acs.est.2c02934. 36134580

[kfae097-B9] Chini M , Hanganu-Opatz IL. 2021. Prefrontal cortex development in health and disease: lessons from rodents and humans. Trends Neurosci. 44(3):227–240. doi: 10.1016/J.TINS.2020.10.017. [accessed 2024 Jan 31]. http://www.cell.com/article/S0166223620302502/fulltext. 33246578

[kfae097-B10] Cleland SE , Wyatt LH, Wei L, Paul N, Serre ML, Jason West J, Henderson SB, Rappold AG. 2022. Short-term exposure to wildfire smoke and PM2.5 and cognitive performance in a brain-training game: a longitudinal study of U.S. adults. Environ Health Perspect. 130(6):67005. doi: 10.1289/EHP10498. [accessed 2023 Apr 13]. https://ehp.niehs.nih.gov/doi/10.1289/EHP10498. 35700064 PMC9196888

[kfae097-B11] Cone DC , MacMillan DS, Van Gelder C, Brown DJ, Weir SD, Bogucki S. 2005. Noninvasive fireground assessment of carboxyhemoglobin levels in firefighters. Prehosp Emerg Care. 9(1):8–13. doi: 10.1080/10903120590891912. [accessed 2024 Jun 19]. https://www.tandfonline.com/doi/abs/10.1080/10903120590891912. 16036821

[kfae097-B12] Dengler-Crish CM , Ball HC, Lin L, Novak KM, Cooper LN. 2018. Evidence of wnt/β-catenin alterations in brain and bone of a tauopathy mouse model of Alzheimer’s disease. Neurobiol Aging. 67:148–158. doi: 10.1016/J.NEUROBIOLAGING.2018.03.021. [accessed 2024 Feb 2]. https://pubmed.ncbi.nlm.nih.gov/29660685/. 29660685

[kfae097-B13] Dillman AA , Majounie E, Ding J, Gibbs JR, Hernandez D, Arepalli S, Traynor BJ, Singleton AB, Galter D, Cookson MR. 2017. Transcriptomic profiling of the human brain reveals that altered synaptic gene expression is associated with chronological aging. Sci Rep. 7(1):16890. doi: 10.1038/S41598-017-17322-0. [accessed 2024 Feb 2]. https://pubmed.ncbi.nlm.nih.gov/29203886/. 29203886 PMC5715102

[kfae097-B14] Eden MJ , Matz J, Garg P, Gonzalez MP, McElderry K, Wang S, Gollner MJ, Oakes JM, Bellini C. 2023. Prolonged smoldering douglas fir smoke inhalation augments respiratory resistances, stiffens the aorta, and curbs ejection fraction in hypercholesterolemic mice. Sci Total Environ. 861:160609. doi: 10.1016/J.SCITOTENV.2022.160609. [accessed 2023 Apr 13]. https://pubmed.ncbi.nlm.nih.gov/36470384/. 36470384 PMC10699119

[kfae097-B15] Gale SD , Erickson LD, Anderson JE, Brown BL, Hedges DW. 2020. Association between exposure to air pollution and prefrontal cortical volume in adults: a cross-sectional study from the UK biobank. Environ Res. 185:109365. doi: 10.1016/J.ENVRES.2020.109365. [accessed 2024 Jan 31]. https://pubmed.ncbi.nlm.nih.gov/32222630/. 32222630

[kfae097-B16] Ganor RS , Harats D, Schiby G, Rosenblatt K, Lubitz I, Shaish A, Salomon O. 2018. Elderly apolipoprotein E-/- mice with advanced atherosclerotic lesions in the aorta do not develop Alzheimer’s disease-like pathologies. Mol Med Rep. 17(2):2488–2492. doi: 10.3892/MMR.2017.8127. [accessed 2024 Jan 30]. https://pubmed.ncbi.nlm.nih.gov/29207114/. 29207114

[kfae097-B17] Garakani K , Shams H, Mofrad MRK. 2017. Mechanosensitive conformation of vinculin regulates its binding to MAPK1. Biophys J. 112(9):1885–1893. doi: 10.1016/J.BPJ.2017.03.039. [accessed 2024 Feb 2]. https://www.ncbi.nlm.nih.gov/pmc/articles/PMC5425409/. 28494959 PMC5425409

[kfae097-B18] Garg P , Roche T, Eden M, Matz J, Oakes Jessica M, Bellini C, Gollner MJ. 2021. Effect of moisture content and fuel type on emissions from vegetation using a steady state combustion apparatus. Int J Wildland Fire. 31(1):14–23. doi: 10.1071/WF20118. PMC8580516 34776721

[kfae097-B19] Ge SX , Son EW, Yao R. 2018. iDEP: an integrated web application for differential expression and pathway analysis of RNA-seq data. BMC Bioinformatics. 19(1):1–24. doi: 10.1186/S12859-018-2486-6. [accessed 2024 Jan 31]. https://bmcbioinformatics.biomedcentral.com/articles/10.1186/s12859-018-2486-6. 30567491 PMC6299935

[kfae097-B20] Genuis SJ , Kelln KL. 2015. Toxicant exposure and bioaccumulation: a common and potentially reversible cause of cognitive dysfunction and dementia. Behav Neurol. 2015:620143. doi: 10.1155/2015/620143. [accessed 2024 Feb 2]. https://pubmed.ncbi.nlm.nih.gov/25722540/. 25722540 PMC4334623

[kfae097-B21] Higuera PE , Cook MC, Balch JK, Stavros EN, Mahood AL, St Denis LA. 2023. Shifting social-ecological fire regimes explain increasing structure loss from Western wildfires. PNAS Nexus. 2(3):pgad005. doi: 10.1093/pnasnexus/pgad005. [accessed 2023 Apr 13]. http://www.ncbi.nlm.nih.gov/pubmed/36938500. 36938500 PMC10019760

[kfae097-B22] Hopkins LE , Laing E, Peake J, Uyeminami D, Mack S, Li X, Smiley-Jewell S, Pinkerton K. 2018. Repeated iron-soot exposure and nose-to-brain transport of inhaled ultrafine particles. Toxicol Pathol. 46(1):75–84. doi: 10.1177/0192623317729222.Repeated. 28914166 PMC6405220

[kfae097-B23] Khayer N , Mirzaie M, Marashi SA, Jalessi M. 2020. Rps27a might act as a controller of microglia activation in triggering neurodegenerative diseases. PLoS One. 15(9):e0239219. doi: 10.1371/JOURNAL.PONE.0239219. [accessed 2024 Feb 2]. https://pubmed.ncbi.nlm.nih.gov/32941527/. 32941527 PMC7498011

[kfae097-B24] Kim YH , Warren SH, Krantz QT, King C, Jaskot R, Preston WT, George BJ, Hays MD, Landis MS, Higuchi M, et al 2018. Mutagenicity and lung toxicity of smoldering vs. Flaming emissions from various biomass fuels: implications for health effects from wildland fires. Environ Health Perspect. 126(1):017011–017014. doi: 10.1289/EHP2200. 29373863 PMC6039157

[kfae097-B25] Koechlin E. 2016. Prefrontal executive function and adaptive behavior in complex environments. Curr Opin Neurobiol. 37:1–6. doi: 10.1016/J.CONB.2015.11.004. [accessed 2024 Jan 31]. https://pubmed.ncbi.nlm.nih.gov/26687618/. 26687618

[kfae097-B26] Kolk SM , Rakic P. 2022. Development of prefrontal cortex. Neuropsychopharmacology. 47(1):41–57. doi: 10.1038/S41386-021-01137-9. [accessed 2024 Jan 31]. https://pubmed.ncbi.nlm.nih.gov/34645980/. 34645980 PMC8511863

[kfae097-B27] Lee HM , Hallberg LM, Greeley GH, Englander EW. 2010. Differential inhibition of mitochondrial respiratory complexes by inhalation of combustion smoke and carbon monoxide, in vivo, in the rat brain. Inhal Toxicol. 22(9):770–777. doi: 10.3109/08958371003770315. [accessed 2024 Feb 2]. https://pubmed.ncbi.nlm.nih.gov/20429857/. 20429857 PMC3398809

[kfae097-B28] Li Y , Han F, Shi Y. 2015. Changes in integrin αv, vinculin and connexin43 in the medial prefrontal cortex in rats under single-prolonged stress. Mol Med Rep. 11(4):2520–2526. doi: 10.3892/MMR.2014.3030. [accessed 2024 Feb 2]. https://pubmed.ncbi.nlm.nih.gov/25483027/. 25483027 PMC4337628

[kfae097-B29] Liu Q , Shkirkova K, Lamorie-Foote K, Connor M, Patel A, Babadjouni R, Huuskonen M, Montagne A, Baertsch H, Zhang H, et al 2021. Air pollution particulate matter exposure and chronic cerebral hypoperfusion and measures of white matter injury in a murine model. Environ Health Perspect. 129(8):87006. doi: 10.1289/EHP8792. [accessed 2024 Feb 2]. https://pubmed.ncbi.nlm.nih.gov/34424052/. 34424052 PMC8382048

[kfae097-B30] Maguschak KA , Ressler KJ. 2012. The dynamic role of beta-catenin in synaptic plasticity. Neuropharmacology. 62(1):78–88. doi: 10.1016/J.NEUROPHARM.2011.08.032. [accessed 2024 Feb 2]. https://pubmed.ncbi.nlm.nih.gov/21903109/. 21903109 PMC3196058

[kfae097-B31] Mohan A , Thalamuthu A, Mather KA, Zhang Y, Catts VS, Weickert CS, Sachdev PS. 2018. Differential expression of synaptic and interneuron genes in the aging human prefrontal cortex. Neurobiol Aging. 70:194–202. doi: 10.1016/j.neurobiolaging.2018.06.011. 30031232

[kfae097-B32] Mokoena ML , Harvey BH, Viljoen F, Ellis SM, Brink CB. 2015. Ozone exposure of flinders sensitive line rats is a rodent translational model of neurobiological oxidative stress with relevance for depression and antidepressant response. Psychopharmacology (Berl). 232(16):2921–2938. doi: 10.1007/S00213-015-3928-8. [accessed 2024 Jan 31]. https://pubmed.ncbi.nlm.nih.gov/25877744/. 25877744

[kfae097-B33] Nativio R , Lan Y, Donahue G, Sidoli S, Berson A, Srinivasan AR, Shcherbakova O, Amlie-Wolf A, Nie J, Cui X, et al 2020. An integrated multi-omics approach identifies epigenetic alterations associated with Alzheimer’s disease. Nat Genet. 52(10):1024–1035. doi: 10.1038/s41588-020-0696-0. [accessed 2024 Feb 2]. https://www.nature.com/articles/s41588-020-0696-0. 32989324 PMC8098004

[kfae097-B34] Navarro KM , Butler CR, Fent K, Toennis C, Sammons D, Ramirez-Cardenas A, Clark KA, Byrne DC, Graydon PS, Hale CR, et al 2021a. The wildland firefighter exposure and health effect (WFFEHE) study: rationale, design, and methods of a repeated-measures study. Ann Work Expo Health. 66(6):714–727. doi: 10.1093/annweh/wxab117. PMC9203592 34919119

[kfae097-B35] Navarro KM , Kleinman MT, Mackay CE, Reinhardt TE, Balmes JR, Broyles GA, Ottmar RD, Naher LP, Domitrovich JW. 2019. Wildland firefighter smoke exposure and risk of lung cancer and cardiovascular disease mortality. Environ Res. 173:462–468. doi: 10.1016/j.envres.2019.03.060. 30981117

[kfae097-B36] Navarro KM , West MR, O’Dell K, Sen P, Chen I-C, Fischer EV, Hornbrook RS, Apel EC, Hills AJ, Jarnot A, et al 2021b. Exposure to particulate matter and estimation of volatile organic compounds across wildland firefighter job tasks. Environ Sci Technol. 55(17):11795–11804. doi: 10.1021/acs.est.1c00847. 34488352 PMC8978153

[kfae097-B37] Oudin A , Segersson D, Adolfsson R, Forsberg B. 2018. Association between air pollution from residential wood burning and dementia incidence in a longitudinal study in Northern Sweden. PLoS One. 13(6):e0198283. doi: 10.1371/JOURNAL.PONE.0198283. 29897947 PMC5999109

[kfae097-B38] Palmeri R , Gupta V. 2023. Carboxyhemoglobin toxicity. Treasure Island (FL): StatPearls. [accessed 2024 Jun 19] https://www.ncbi.nlm.nih.gov/books/NBK557888/. 32491811

[kfae097-B39] Palomer E , Buechler J, Salinas PC. 2019. Wnt signaling deregulation in the aging and Alzheimer’s brain. Front Cell Neurosci. 13:227. doi: 10.3389/FNCEL.2019.00227. [accessed 2024 Feb 2]. https://pubmed.ncbi.nlm.nih.gov/31191253/. 31191253 PMC6538920

[kfae097-B40] Pelletier C , Ross C, Bailey K, Fyfe TM, Cornish K, Koopmans E. 2022. Health research priorities for wildland firefighters: a modified delphi study with stakeholder interviews. BMJ Open. 12(2):e051227. doi: 10.1136/BMJOPEN-2021-051227. [accessed 2024 Jan 30]. https://pubmed.ncbi.nlm.nih.gov/35115350/. PMC8814744 35115350

[kfae097-B41] Perluigi M , Barone E, Di Domenico F, Butterfield DA. 2016. Aberrant protein phosphorylation in Alzheimer disease brain disturbs pro-survival and cell death pathways. Biochim Biophys Acta. 1862(10):1871–1882. doi: 10.1016/J.BBADIS.2016.07.005. 27425034

[kfae097-B42] Peters A , Veronesi B, Calderón-Garcidueñas L, Gehr P, Chen LC, Geiser M, Reed W, Rothen-Rutishauser B, Schürch S, Schulz H. 2006. Translocation and potential neurological effects of fine and ultrafine particles a critical update. Part Fibre Toxicol. 3:13. doi: 10.1186/1743-8977-3-13. [accessed 2024 Jan 30]. https://pubmed.ncbi.nlm.nih.gov/16961926/. 16961926 PMC1570474

[kfae097-B43] Petkus AJ , Resnick SM, Wang X, Beavers DP, Espeland MA, Gatz M, Gruenewald T, Millstein J, Chui HC, Kaufman JD, et al 2022. Ambient air pollution exposure and increasing depressive symptoms in older women: the mediating role of the prefrontal cortex and insula. Sci Total Environ. 823:153642. doi: 10.1016/J.SCITOTENV.2022.153642. 35122843 PMC8983488

[kfae097-B44] Puig MV , Gulledge AT. 2011. Serotonin and prefrontal cortex function: neurons, networks, and circuits. Mol Neurobiol. 44(3):449–464. doi: 10.1007/S12035-011-8214-0. [accessed 2024 Jan 31]. https://pubmed.ncbi.nlm.nih.gov/22076606/. 22076606 PMC3282112

[kfae097-B45] Ramos C , Cañedo-Mondragón R, Becerril C, González-ávila G, Esquivel AL, Torres-Machorro AL, Montaño M. 2021. Short-term exposure to wood smoke increases the expression of pro-inflammatory cytokines, gelatinases, and TIMPs in guinea pigs. Toxics. 9(9):227. doi: 10.3390/TOXICS9090227. 34564378 PMC8473192

[kfae097-B46] Reid CE , Brauer M, Johnston FH, Jerrett M, Balmes JR, Elliott CT. 2016. Critical review of health impacts of wildfire smoke exposure. Environ Health Perspect. 124(9):1334–1343. doi: 10.1289/EHP.1409277. [accessed 2023 Apr 13]. https://pubmed.ncbi.nlm.nih.gov/27082891/. 27082891 PMC5010409

[kfae097-B47] Reid CE , Maestas MM. 2019. Wildfire smoke exposure under climate change: impact on respiratory health of affected communities. Curr Opin Pulm Med. 25(2):179–187. doi: 10.1097/MCP.0000000000000552. 30461534 PMC6743728

[kfae097-B48] Rocha SM , Bantle CM, Aboellail T, Chatterjee D, Smeyne RJ, Tjalkens RB. 2022. Rotenone induces regionally distinct α-synuclein protein aggregation and activation of glia prior to loss of dopaminergic neurons in C57Bl/6 mice. Neurobiol Dis. 167:105685. doi: 10.1016/J.NBD.2022.105685. [accessed 2024 Feb 2]. https://pubmed.ncbi.nlm.nih.gov/35257879/. 35257879 PMC9615439

[kfae097-B49] Rubin ES , Parker PB, Garg B, Wu D, Peregrine J, Lee D, Amato P, Gibbins KJ, Baldwin MK, O’Leary T, et al 2021. Wildfire smoke exposure is associated with decreased total motile sperm count. Fertil Steril. 116(3):e89. doi: 10.1016/j.fertnstert.2021.07.248. 10.1016/j.fertnstert.2021.07.248. 38346549

[kfae097-B50] Ruby BC , Coker RH, Sol J, Quindry J, Montain SJ. 2023. Physiology of the wildland firefighter: managing extreme energy demands in hostile, smoky, mountainous environments. Compr Physiol. 13(2):4587–4615. doi: 10.1002/CPHY.C220016. [accessed 2024 Jan 30]. https://onlinelibrary.wiley.com/doi/full/10.1002/cphy.c220016. 36994767

[kfae097-B51] Schnell T , Suhr F, Weierstall-Pust R. 2020. Post-traumatic stress disorder in volunteer firefighters: influence of specific risk and protective factors. Eur J Psychotraumatol. 11(1):1764722. doi: 10.1080/20008198.2020.1764722. 33029308 PMC7473037

[kfae097-B52] Schuller A , Bellini C, Jenkins TG, Eden M, Matz J, Oakes J, Montrose L. 2021. Simulated wildfire smoke significantly alters sperm dna methylation patterns in a murine model. Toxics. 9(9):199. doi: 10.3390/TOXICS9090199/S1. [accessed 2023 Feb 3]. https://www.mdpi.com/2305-6304/9/9/199/htm. 34564350 PMC8473101

[kfae097-B53] Schuller A , Montrose L. 2020. Influence of woodsmoke exposure on molecular mechanisms underlying Alzheimer’s disease: existing literature and gaps in our understanding. Epigenet Insights. 13:2516865720954873. doi: 10.1177/2516865720954873. 32974607 PMC7493275

[kfae097-B54] Scieszka D , Hunter R, Begay J, Bitsui M, Lin Y, Galewsky J, Morishita M, Klaver Z, Wagner J, Harkema JR, et al 2022. Neuroinflammatory and neurometabolomic consequences from inhaled wildfire smoke-derived particulate matter in the Western United States. Toxicol Sci. 186(1):149–162. doi: 10.1093/toxsci/kfab147. 34865172 PMC8883349

[kfae097-B55] Scieszka D , Jin Y, Noor S, Barr E, Garcia M, Begay J, Herbert G, Hunter RP, Bhaskar K, Kumar R, et al 2023. Biomass smoke inhalation promotes neuroinflammatory and metabolomic temporal changes in the hippocampus of female mice. J Neuroinflammation. 20(1):1–15. doi: 10.1186/S12974-023-02874-Y/FIGURES/5. [accessed 2024 Jan 31]. https://jneuroinflammation.biomedcentral.com/articles/10.1186/s12974-023-02874-y. 37608305 PMC10464132

[kfae097-B56] Semmens EO , Leary CS, West MR, Noonan CW, Navarro KM, Domitrovich JW. 2021. Carbon monoxide exposures in wildland firefighters in the United States and targets for exposure reduction. J Expo Sci Environ Epidemiol. 31(5):923–929. doi: 10.1038/S41370-021-00371-Z. [accessed 2024 Jun 19]. https://pubmed.ncbi.nlm.nih.gov/34285366/. 34285366 PMC8448930

[kfae097-B57] Sosedova LM , Vokina VA, Novikov MA, Zhurba OM, Alekseenko AN, Rukavishnikov VS, Andreeva ES. 2021. Paternal biomass smoke exposure in rats produces behavioral and cognitive alterations in the offspring. Toxics. 9(1):1–11. doi: 10.3390/TOXICS9010003. PMC7823662 33396546

[kfae097-B58] Stanley IH , Hom MA, Gai AR, Joiner TE. 2018. Wildland firefighters and suicide risk: examining the role of social disconnectedness. Psychiatry Res. 266:269–274. doi: 10.1016/J.PSYCHRES.2018.03.017. [accessed 2024 Jan 31]. https://pubmed.ncbi.nlm.nih.gov/29573853/. 29573853

[kfae097-B59] Supek F , Bošnjak M, Škunca N, Šmuc T. 2011. REVIGO summarizes and visualizes long lists of gene ontology terms. PLoS One. 6(7):e21800. doi: 10.1371/JOURNAL.PONE.0021800. [accessed 2024 Jan 31]. https://journals.plos.org/plosone/article?id=10.1371/journal.pone.0021800. 21789182 PMC3138752

[kfae097-B60] Thangavel P , Park D, Lee YC. 2022. Recent insights into particulate matter (PM2.5)-mediated toxicity in humans: an overview. Int J Environ Res Public Health. 19(12). doi: 10.3390/IJERPH19127511. [accessed 2024 Jan 30]. https://pubmed.ncbi.nlm.nih.gov/35742761/. PMC9223652 35742761

[kfae097-B61] Tomei F , Rosati MV, Ciarrocca M, Baccolo TP. 2004. Occupational exposure to urban pollutants and urinary 5-hydroxy-3-indoleacetic acid—PubMed. J Environ Health. 66(6):38–42. [accessed 2024 Jan 31]. https://pubmed.ncbi.nlm.nih.gov/14768281/. 14768281

[kfae097-B62] Tooley UA , Bassett DS, Mackey AP. 2021. Environmental influences on the pace of brain development. Nat Rev Neurosci. 22(6):372–384. doi: 10.1038/s41583-021-00457-5. [accessed 2024 Jan 31]. https://www.nature.com/articles/s41583-021-00457-5. 33911229 PMC8081006

[kfae097-B63] Turco M , Abatzoglou JT, Herrera S, Zhuang Y, Jerez S, Lucas DD, AghaKouchak A, Cvijanovic I. 2023. Anthropogenic climate change impacts exacerbate summer forest fires in California. Proc Natl Acad Sci USA. 120(25):e2213815120. doi: 10.1073/PNAS.2213815120/SUPPL_FILE/PNAS.2213815120.SAPP.PDF. [accessed 2024 Jan 30]. https://www.pnas.org/doi/abs/10.1073/pnas.2213815120. 37307438 PMC10288651

[kfae097-B64] Wang Q , Huang X, Su Y, Yin G, Wang S, Yu B, Li H, Qi J, Chen H, Zeng W, et al 2022. Activation of wnt/β-catenin pathway mitigates blood–brain barrier dysfunction in Alzheimer’s disease. Brain. 145(12):4474–4488. doi: 10.1093/BRAIN/AWAC236. [accessed 2024 Feb 2]. 10.1093/BRAIN/AWAC236. 35788280 PMC9762951

[kfae097-B65] Wen J , Burke M. 2022. Lower test scores from wildfire smoke exposure. Nat Sustain. 5(11):947–955. doi: 10.1038/s41893-022-00956-y. [accessed 2023 Apr 13]. https://www.nature.com/articles/s41893-022-00956-y.

[kfae097-B66] Wu CM , Adetona O, Song C. 2021. Acute cardiovascular responses of wildland firefighters to working at prescribed burn. Int J Hyg Environ Health. 237:113827. doi: 10.1016/J.IJHEH.2021.113827. 34403889

[kfae097-B67] Xu P , Chen A, Li Y, Xing X, Lu H. 2019. Medial prefrontal cortex in neurological diseases. Physiol Genomics. 51(9):432–442. doi: 10.1152/PHYSIOLGENOMICS.00006.2019. [accessed 2024 Jan 31]. https://pubmed.ncbi.nlm.nih.gov/31373533/. 31373533 PMC6766703

[kfae097-B68] Yamashita CM , Fessler MB, Vasanthamohan L, Lac J, Madenspacher J, McCaig L, Yao L, Wang L, Puntorieri V, Mehta S, et al 2014. Apolipoprotein E-deficient mice are susceptible to the development of acute lung injury. Respiration. 87(5):416–427. doi: 10.1159/000358438. [accessed 2024 Jan 30]. 10.1159/000358438. 24662316 PMC4659709

[kfae097-B69] Yokota S , Oshio S, Moriya N, Takeda K. 2016. Social isolation-induced territorial aggression in male offspring is enhanced by exposure to diesel exhaust during pregnancy. PLoS One. 11(2):e0149737. doi: 10.1371/JOURNAL.PONE.0149737. [accessed 2024 Jan 31]. https://pubmed.ncbi.nlm.nih.gov/26919122/. 26919122 PMC4769130

[kfae097-B70] Zhang B , Weuve J, Langa KM, D’Souza J, Szpiro A, Faul J, Mendes de Leon C, Gao J, Kaufman JD, Sheppard L, et al 2023. Comparison of particulate air pollution from different emission sources and incident dementia in the US. JAMA Intern Med. 183(10):1080–1089. doi: 10.1001/JAMAINTERNMED.2023.3300. [accessed 2024 Feb 2]. https://jamanetwork.com/journals/jamainternalmedicine/fullarticle/2808088. 37578757 PMC10425875

