# Supplementary material for: Robust differential gene expression patterns in the prefrontal cortex of male mice exposed to an occupationally relevant dose of laboratory-generated wildfire smoke
Source: Toxicol Sci. 2024 Aug 6;201(2):300–10. doi: 10.1093/toxsci/kfae097 (PMC11424885; doi:10.1093/toxsci/kfae097)
Supplement: kfae097_Supplementary_Data [file kfae097_supplementary_data.docx]

**Supplemental Figures:**

**Robust differential gene expression patterns in the pre-frontal cortex of male mice exposed to an occupationally relevant dose of laboratory generated wildfire smoke**

Adam Schuller,^1^ Jessica Oakes,^2^ Tom LaRocca,^3^ Jacqueline Matz,^2^ Matthew Eden,^2^ Chiara Bellini,^2^ and Luke Montrose^1,a^

*^1^ Department of Environmental and Radiological Health Sciences, Colorado State University, Fort Collins, CO, USA*

*^2^ Department of Bioengineering, Northeastern University, Boston, MA, USA*

*^3^ Department of Health and Exercise Science, Colorado State University, Fort Collins, CO, USA*

*^a^ corresponding author*

**Corresponding author e-mail:**

luke.montrose@colostate.edu

**Corresponding author address:**

350 W Lake St

Fort Collins, CO 80538

**Keywords:**

wildfire smoke, wildland firefighter, neurotoxicology, occupational exposure, RNAseq

**Running title:**

Neurotoxic transcriptomic effects of wildland firefighting

**Figure S1. A)** Top 20 Kyoto encyclopedia of genes and genomes (KEGG) pathways between smoke exposed and filtered air control samples (FDR < 0.05) across all 1,396 downregulated DEGs identified in our dataset. **B)** Top 20 KEGG pathways between smoke exposed and filtered air control samples (FDR < 0.05) across all 1,466 upregulated DEGs identified in our dataset. **C)** Top 20 Reactome pathways between smoke exposed and filtered air control samples (FDR < 0.05) across all 1,396 downregulated DEGs identified in our dataset. **D)** Top 20 Reactome pathways between smoke exposed and filtered air control samples (FDR < 0.05) across all 1,466 upregulated DEGs identified in our dataset. For all panels, bubble size represents gene count while color corresponds to adjusted p-value (Benjamini and Hochberg method).

**Figure S2.** Neuroinflammation-relevant panel of genes/transcripts that show the same directionality as a previous publication^21^ assessing wildfire smoke exposure in the same brain region (PFC) of male mice. Although the dose, duration, and combustion parameters vary, there is still congruence in the significance of expression between smoke exposed and filtered air control groups. Each bar represents the mean FPKM with error bars demonstrating standard deviation. Value over each comparison represents FDR.
